# Malaria ookinetes exhibit multiple markers for apoptosis-like programmed cell death *in vitro*

**DOI:** 10.1186/1756-3305-2-32

**Published:** 2009-07-15

**Authors:** Shashini C Arambage, Karen M Grant, Ian Pardo, Lisa Ranford-Cartwright, Hilary Hurd

**Affiliations:** 1 Institute of Science and Technology in Medicine, Centre for Applied Entomology and Parasitology, School of Life Sciences, Huxley Building, Keele University, Staffordshire, ST5 5BG, UK; 2 School of Health & Medicine, Faraday Building, Lancaster University, Lancaster, LA1 4YB, UK; 3 Division of Infection & Immunity, Faculty of Biomedical & Life Sciences, University of Glasgow, Glasgow Biomedical Research Centre, 120 University Place, Glasgow G12 8TA, UK

## Abstract

**Background:**

A wide range of unicellular eukaryotes have now been shown to undergo a form of programmed cell death (PCD) that resembles apoptosis; exhibiting morphological and, in some cases, biochemical markers typical of metazoans. However, reports that sexual and asexual stages of malaria parasites exhibit these markers have been challenged. Here we use a rodent malaria model, *Plasmodium berghei*, to determine whether, and what proportion of cultured ookinetes show signs of apoptosis-like death and extend the study to examine ookinetes of *Plasmodium falciparum in vivo*.

**Results:**

Ookinetes displayed the following markers of PCD: loss of mitochondrial membrane potential, nuclear chromatin condensation, DNA fragmentation, translocation of phosphatidylserine to the outer surface of the cell membrane and caspase-like activity. The proportion of parasites expressing apoptosis markers rose with time, particularly when cultured in phosphate buffered saline. Some ookinetes positive for apoptosis markers also had compromised membranes, which could represent a late stage in the process. When these are included a similar proportion of ookinetes display each marker. Over 50% of *P. falciparum *ookinetes, removed from the mosquito midgut lumen 24 h post-infection, had nuclei containing fragmented DNA.

**Conclusion:**

We have confirmed previous reports that *Plasmodium *ookinetes display multiple signs that suggest they die by a mechanism resembling apoptosis. This occurs *in vivo *and *in vitro *without experimental application of triggers. Our findings support the hypothesis that non-necrotic mechanisms of cell death evolved before the advent of multicellular organisms.

## Background

The hypothesis that a protozoan parasite can undergo a form of programmed cell death (PCD) akin to apoptosis was first proposed by Ameisen and colleagues in 1995 [1]. Since then reports of unicellular eukaryotes displaying markers of PCD have been accumulating steadily and Deponte has recently pointed out that unicellular organism from all of the major eukaryote taxa bar one have been shown to display one or more classical apoptotic marker during cell death [2]. These organisms include two much studied models, the yeast *Saccharomyces cerevisiae *[3] and the slime mould, *Dictyostelium discoideum *[4]. Amongst the parasitic protists, the trypanosomatids have been the focus of most research on PCD [5-11], but PCD features have also been recorded in;*Toxoplasma gondii *[12]; *Blastocystis hominis *[13]; in protozoans that do not possess mitochondria such as *Giardia intestinalis, Trichomonas foetus *and *T. vaginalis *[14,15]*; *and in *P. berghei *and *P. falciparum *[16,17].

Increasing knowledge of the morphological appearance and biochemical pathways of cell death in metazoans has led to an acceptance that the classification into necrosis, apoptosis and autophagy is too simplistic and that, in some circumstances, different aspects of these death pathways may be combined and stereotypic patterns do not always apply [18]. The majority of the parasites listed above display several independent morphological markers that are associated with typical mammalian apoptotic phenotypes, including the collapse of chromatin into condensed electron-dense masses; nuclear fragmentation; a change in plasma membrane permeability and the production of apoptotic bodies. The accompanying biochemical markers include a loss of mitochondrial membrane potential Δψ_m; _cytochrome c release into the cytoplasm; activation of caspase-like molecules; translocation of phosphatidylserine (PS) to the outer leaflet of the plasma membrane; and DNA fragmentation [2,19,20]. Both biochemical and morphological markers can be detected in protozoans that have been exposed to a variety of stressful stimuli including starvation; heat shock; incubation with nitric oxide donors or reactive oxygen species; and treatment with staurosporine or anti-parasitic drugs. They also occur in stationary phase parasite cultures and in differentiating forms in vector insects [2,20]. However, there is considerable variation both in the kind and number of markers detected in different species and in which stimuli induce which markers. In addition, features associated with autophagy have also been described [21] and it is by no means clear whether PCD in parasitic protists should be classified as apoptosis or whether new terminology is needed.

Few studies have so far been made of PCD in *Plasmodium *spp. and the majority of these have focussed on the erythrocytic stages of the human malaria, *P. falciparum*, where conflicting observations and conclusions have been made [2,17,22]. The occurrence of apoptosis-like cell death has also been disputed in the rodent malaria *P. berghei*. Al-Olayan *et al*. [23,24] reported multiple features of apoptosis-like cell death in ookinetes *in vivo*, in the mosquito midgut lumen, and *in vitro*. In contrast, Le Chat and colleagues [25] found no evidence for the occurrence of nuclear chromatin condensation or DNA fragmentation in *P. berghei *ookinetes grown in culture. In addition, they only observed a very low number of ookinetes displaying phosphatidylserine translocation or activated caspase-like molecules.

In view of this discrepancy in observations we have examined *P. berghei *ookinetes grown in culture to assess the proportion of a parasite population that displays multiple morphological and biochemical markers that have been associated with mammalian apoptosis. Changes in the percentage of parasites dying by an apoptosis-like process over time, and in different culture conditions, have been assessed. In contrast to most studies of PCD in protozoan parasites, no specific triggers have been used to induce cell death as it occurs naturally in our standard culture conditions. Our results confirm that several markers associated with apoptotic cell death are displayed by ookinetes and that the proportion of parasites exhibiting these markers increases with time. In addition, we demonstrated that *P. falciparum *ookinetes also undergo apoptosis in the mosquito.

## Results

### Markers for apoptosis

#### Condensed chromatin

**Figure 1:**
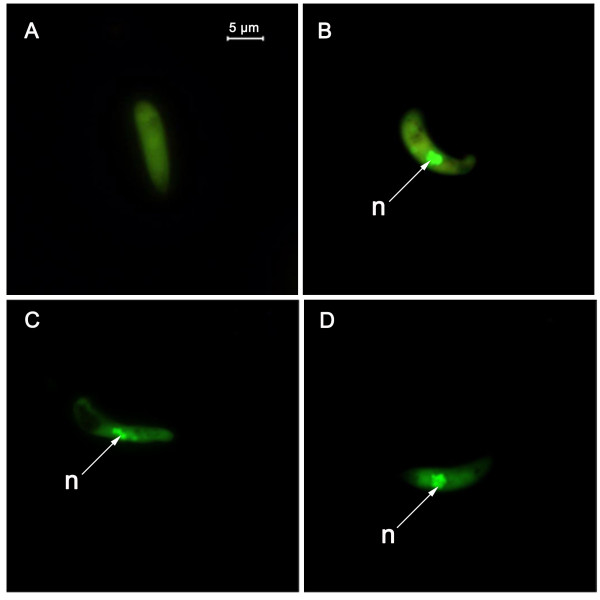
**Ookinetes stained to show nuclear condensation and fragmentation**. *P. berghei *ookinetes were stained with acridine orange. (A) A live ookinete without condensed chromatin. (B) Apoptotic ookinete staining for condensed chromatin (C, D) ookinetes with a fragmented nucleus and condensed chromatin. n: Nucleus.

**Figure 2:**
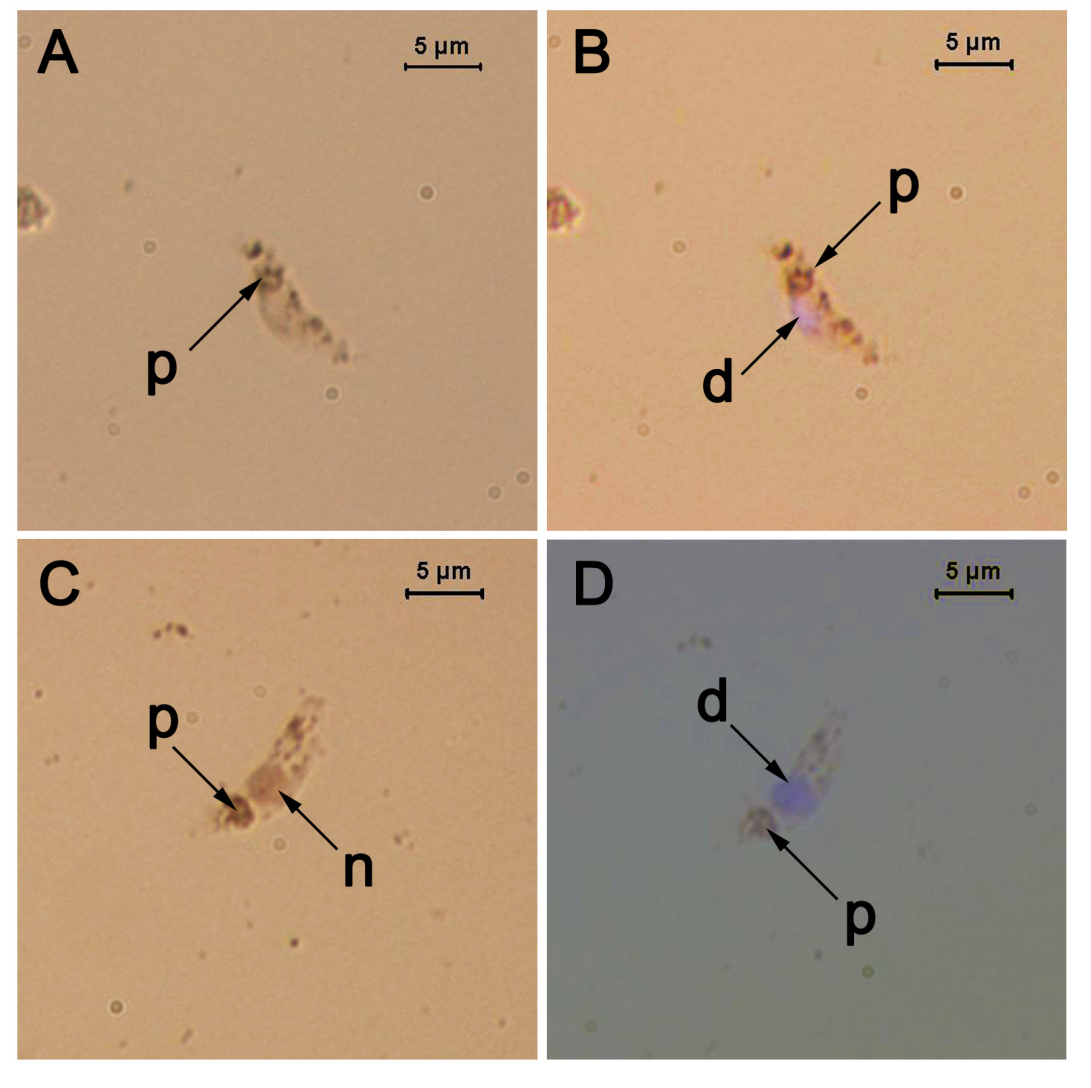
**Detection of fragmented DNA in *P. berghei *ookinetes**. *P. berghei *ookinetes were fixed, subjected to a TUNEL histochemical assay and counter stained with DAPI. n = nucleus stained with TUNEL, d = nucleus counter stained with DAPI, p = the malaria pigment, haemazoin. A and C are images taken under bright light, B is an image of A taken under fluorescent light and D is a merged image of C taken under light and fluorescence.

Acridine orange staining of ookinetes, collected after incubating gametocytaemic blood for 18 h, demonstrated that a proportion of the population contained nuclei with condensed chromatin. These proportions were highly variable from experiment to experiment and with time post-incubation (see Fig. 1 and 2). In a few cases fragmentation of the nucleus could be observed (Fig. 1C, D).

#### Fragmented DNA

Ookinetes collected at the same time as those used for acridine orange staining also displayed signs of DNA fragmentation, as detected using a TUNEL assay (Fig 2: 48.55 ± 6.01%, 64.19 ± 6.09% and 69.89 ± 2.81% at 18, 22 and 26 h post-incubation respectively). A negative control for the TUNEL assay (without TdT enzyme) was conducted to detect the amount of non-specific staining associated with the assay, which would result in false positives. A proportion of ookinetes was positive for TUNEL staining in the presence of TdT and a much smaller proportion was stained in the absence of TdT, indicating that approximately 10% of TUNEL positive ookinetes were non-specifically stained and did not contain fragmented DNA. This was not significantly different from controls at 18, 22 and 26 h post-incubation (p = 0.8015, p = 0.7921, p = 3.001 at 18, 22 and 26 h respectively).

#### Caspase-like activity

**Figure 3:**
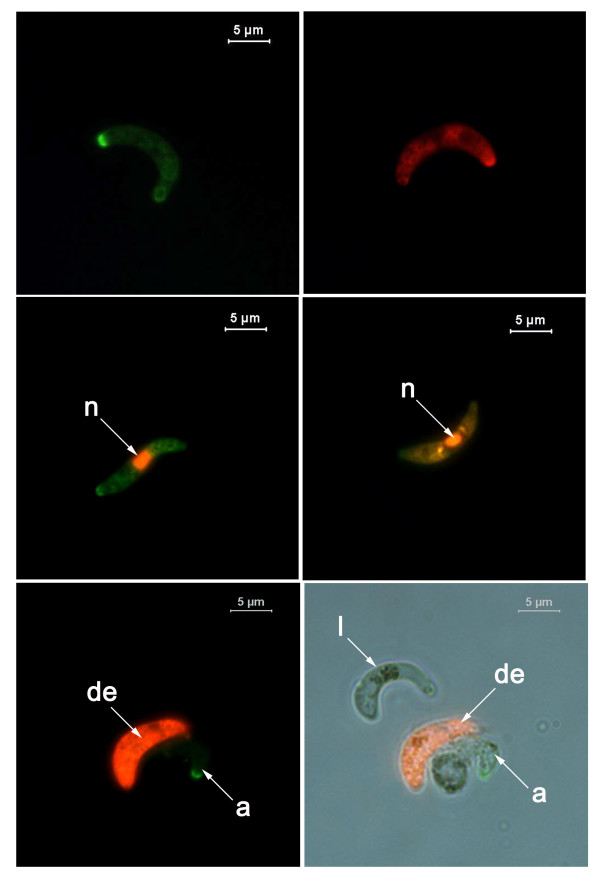
**Detection of caspase-like activity in the cytoplasm of *P. berghei *ookinetes**. *P. berghei *ookinetes were subjected to a pancaspase CaspaTag assay, during which the fluorochrome inhibitor of caspases; FAM.VAD.fmk (green in A, C, D, E and F) or sulforhodamine conjugated caspase inhibitor; SR.DEVD.fmk (red in B) bound to activated caspase-like molecules. Cells with compromised cell membranes stain with propidium iodide (PI, orange). Ookinetes that show both caspase-like activity and membrane integrity are categorised as early apoptotic ookinetes (Green in A). Late apoptotic/dying ookinetes show caspase-like activity and loss of membrane integrity at the same time (C and D). Cells with compromised cell membranes, but no caspase activity were categorised as dead ookinetes (orange in E and F). F is a merged image of E, taken under bright field and FITC filter. Live ookinetes do not show caspase-like activity (see E and F). n: nucleus, de:dead, a:apoptotic, l:live.

Activity of caspase-like molecules was detected using both FAM.VAD.fmk (green) and SR.DEVD.fmk (red) (Fig. 3). A pilot study was conducted to determine the effect of incubation with FAM.VAD.fmk at the manufacturer's recommended temperature of 37°C compared to 19°C, a temperature at which *P. berghei *ookinetes will develop successfully. Ookinetes were collected at 22 h post-incubation, assayed for caspase-like activity and counterstained with propidium iodide (PI) immediately prior to examination to identify those with permeable membranes. The experiment was repeated 6 times and 150 ookinetes examined each time. A significant increase (from 12.36 ± 1.94% to 20.5 ± 3.07%; p = 0.016) in the proportion of cells that had permeable membranes occurred when the assay was performed at 37°C, whereas the proportion of ookinetes displaying caspase-like activity did not change (~42%). All subsequent CaspaTag assays were conducted at 19°C in case the death of some of the ookinetes scored positive at 37°C occurred due to the inappropriate temperature used for the assay incubation.

In cases where FAM.VAD.fmk was used together with PI, the following grouping method was used consistently throughout experiments. Ookinetes that did not show caspase-like activity were considered live. Ookinetes that expressed both caspase-like activity and intact cell membranes (green) were categorised as early apoptotic ookinetes. The small proportion of ookinetes that showed both caspase-like activity and loss of membrane integrity at the same time (green and orange) were grouped as late apoptotic/dying ookinetes. Ookinetes with compromised cell membranes, but no caspase activity (entirely orange), were considered dead.

#### Translocation of phosphatidylserine

**Figure 4:**
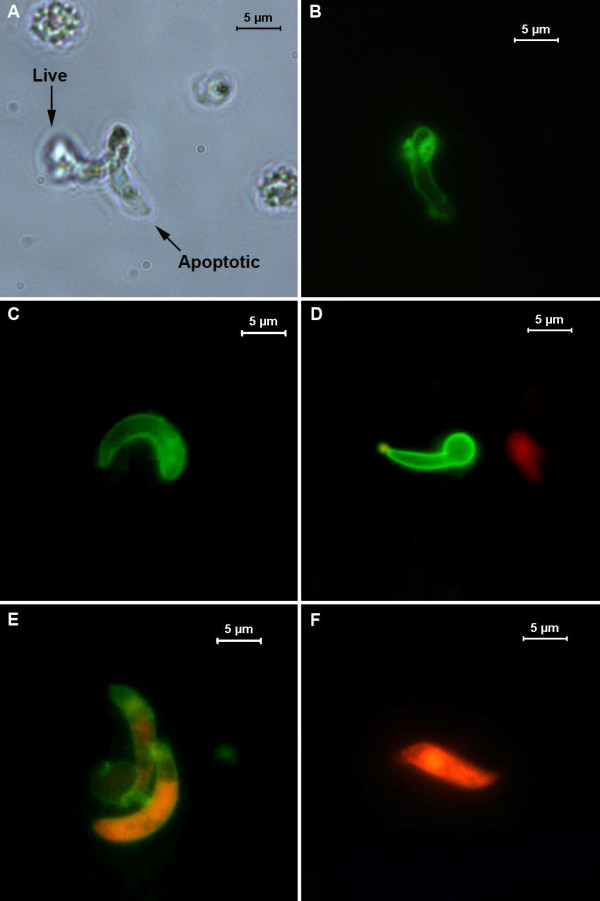
**Detection of translocation of phosphatidylserine to the outer surface of the plasma membrane**. *P. berghei *ookinetes were subjected to an Annexin V assay. Live ookinetes do not label with annexin V, whilst the outer margin of apoptotic ookinetes appears green under FITC filter. A and B are the same field of view, taken under bright field and FITC filter respectively. Live (A) and apoptotic ookinetes (B and C), apoptotic retort-form ookinete (D), late apoptotic ookinetes stained with both annexin (green) and propidium iodide (orange) stains (E), dead ookinetes stain only with propidium iodide (F).

Apoptotic ookinetes, which had translocated their phosphatidylserine (PS) to the outside of the cell membrane, displayed green annexin labelling on the cell surface (Fig. 4A–D). Ookinetes with no labelling were considered live whereas ookinetes that appeared orange throughout (PI permeable), with no annexin labeling, were considered dead. Some ookinetes were labelled yellow-red throughout the cytoplasm, had red nuclear staining and green labeling of the outer surface of the cell (Fig 4E, F). The fate of these cells was uncertain. They could be ookinetes that had developed compromised plasma membranes at a late stage of apoptosis; or they could represent cell with a compromised cell membrane that had died by necrosis, in which annexin had penetrated the cell and labeled PS that was still located on the inner surface of the plasma membrane. In view of this uncertainty, ookinetes with this type of staining were not counted as being apoptotic (for example; 16.23 ± 4.19% annexin positive, 13.32 ± 0.727% annexin and PI positive and 14.87 ± 2.55% PI positive ookinetes at 18 h post-incubation).

#### Mitochondrial membrane potential

**Figure 5:**
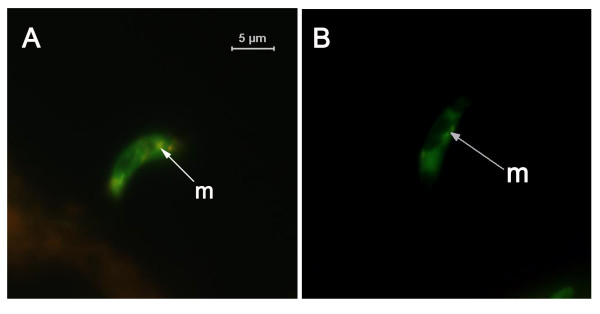
**Identification of mitochondria demonstrating loss of membrane potential**. *P. berghei *ookinetes were subjected to a JC-1 assay to detect MOMP. (A) A normal ookinete with accumulation of orange aggregates, (B) An ookinete with loss of orange aggregates, m: mitochondria.

Ookinetes were collected from ookinete medium after incubating gametocytaemic blood for 18 h and the proportion that exhibited mitochondria outer membrane permeability (MOMP) was determined. The number of ookinetes that did not contain orange fluorescent JC-1 aggregates in their mitochondrion, and thus exhibited mitochondria outer membrane permeability (MOMP) was determined. This experiment was performed on different samples of ookinetes from those examined for other markers of apoptosis. A mean proportion of 34.38 ± 2.95% of ookinetes exhibited MOMP (mean of 3 experiments, 100 ookinetes examined per experiment) (Fig. 5).

### Signs of apoptosis in *P. falciparum *ookinetes

**Figure 6:**
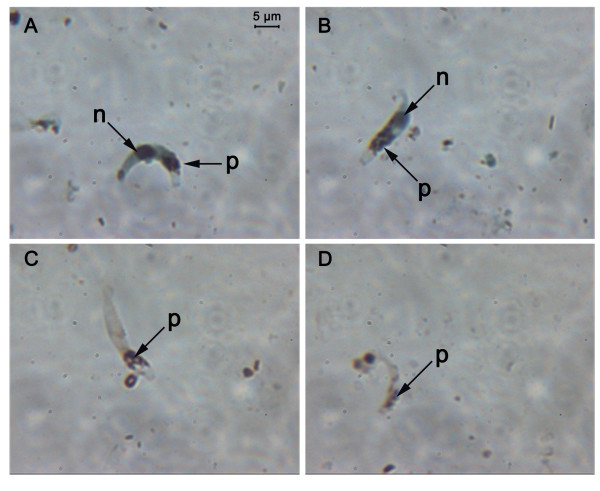
**Ookinetes of *Plasmodium falciparum *assayed for fragmented DNA**. *P. falciparum *ookinetes that had developed in vivo were subjected to a TUNEL assay. A, B: ookinetes showing +ve staining for DNA fragmentation. C, D: ookinetes negative for fragmented DNA. n: nucleus, p: malaria pigment, haemazoin.

A total of 12 *An. gambiae *midguts were examined 24 h post-infection with *P. falciparum*. All midguts contained *P. falciparum *ookinetes with numbers ranging from 18–110 per midgut (mean = 60.24 ± 29.01), of which a mean of 67.8% ± 2.82 were TUNEL positive (see Fig. 6).

### A temporal profile of the expression of apoptosis markers

One problem that was encountered was how to classify ookinetes that displayed markers for apoptosis but also had compromised membranes. This was particularly problematical for detection of PS translocation by annexin labelling, as discussed above. Thus, for the annexin label, PI positive cells were not regarded as apoptotic. In contrast, staining for apoptotic markers was regarded as a positive indication of apoptosis for the other tests, regardless of whether the cell membrane was compromised or not because conclusions regarding labeling were not affected by PI influx.

**Figure 7:**
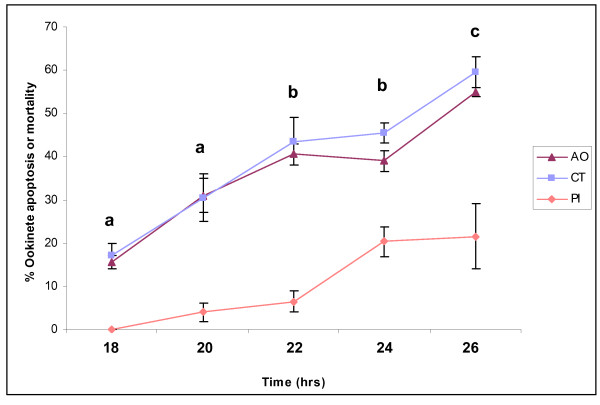
**A temporal profile of apoptosis markers displayed by ookinetes cultured in phosphate buffered saline**. *P. berghei *ookinetes were collected after incubating gametocytaemic blood for 17 h and resuspended in PBS at 18 h. The proportion of apoptotic or dead cells out of 100 ookinetes was detected using acridine orange (AO: nuclear chromatin condensation), CaspaTag (CT: caspase activity) tests, at 5 different time points. Ookinetes with compromised membranes, and no detectable apoptosis markers were detected using propidium iodide(PI). Data represents a mean of 2 independent experiments, n = 100 per sample. Error bars: ± SEM. Different letters show significant differences in apoptosis from 18 h.

A temporal profile of the display of condensed chromatin and caspase-activity was constructed by removing aliquots from the ookinete/PBS suspension at various time points post-blood collection. Overall both markers of apoptosis increased significantly with time (F_(4,10) _= 37.42; p < 0.001). Nuclear chromatin condensation and activation of caspase-like molecules were seen in 15.5 ± 1.06 and 17.0 ± 2.12% of ookinetes respectively at 18 h (1 h post-collection), and by 22 h and 26 h post-blood collection a significant increase in the proportion of ookinetes exhibiting both markers was observed (Tukey's pairwise comparisons, acridine orange: p = 0.0113 and p = 0.0003 at 22 and 26 hrs respectively; CaspaTag: p = 0.0075 and p = 0.0002 at 22 and 26 hrs respectively; Fig. 7). There was no significant difference between the proportion of ookinetes exhibiting markers for nuclear condensation and those that demonstrated activation of caspase-like molecules at any time point (2 way ANOVA, F_(1,10) _= 1.81, p = 0.208). The proportion of ookinetes with compromised membranes and no observable caspase staining (dead) was also observed to rise over time but the rate of increase was not as great as that observed with the markers of apoptosis.

**Figure 8:**
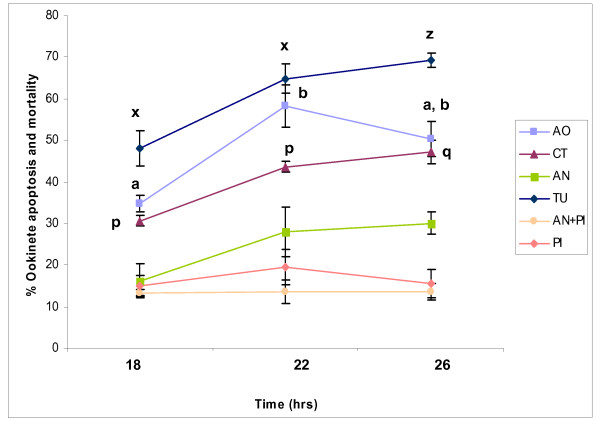
**A temporal profile of the proportion of ookinetes exhibiting several markers of apoptosis when cultures in ookinete medium**. Gametocytaemic blood was incubated at 19°C for varying times. Ookinetes from the same sample were purified at 3 different time points, 18, 22 and 26 h post-blood collection and checked for 4 markers of apoptosis condensed chromatin (using acridine orange: AO), caspase activity (using CaspaTag: CT), phosphatidylserine translocation (using annexin V: AN) and DNA fragmentation (using TUNEL: TU). Ookinetes staining with propidium iodide alone were regarded as dead: PI,: ookinetes showing annexin and PI staining were regarded as at a late stage of apoptosis: AN+PI. Points are means of 3 independent experiments, n = 150 per sample. The proportion of ookinetes displaying three of the markers increased significantly with time and for each of these markes different letters show significant differences from the proportion displaying this marker at 18 h, error bars: ± SEM.

In addition, gametocytaemic blood samples were incubated at 19°C for 18, 22 or 26 h and immediately checked for 4 markers of apoptosis, namely; nuclear chromatin condensation (acridine orange), activation of caspase-like molecules (CaspaTag), translocation of phosphatidylserine (annexin V) and nuclear chromatin fragmentation (TUNEL). Ookinetes were incubated in supplemented RPMI (ookinete medium) throughout. A significant increase in the proportions of ookinetes exhibiting markers of apoptosis was observed from 18 h (2 way ANOVA) (Fig. 8 and Table 1). Double the number of ookinetes with condensed chromatin (34.5 ± 1.76%) and activated caspase-like molecules (30.15 ± 2.14%) was observed at 18 h compared to the previous experiments and a large variation between experimental replicates occurred at almost all time points. Overall significant differences were observed between different markers of apoptosis (separating those with compromised membranes) (2 way ANOVA, F_(4,129) _= 132.04; p = 0.001), and a significant interaction was observed between both time and markers (F_(8,129) _= 2.96; p = 0.005). Tukey's paired comparisons showed that the proportion of ookinetes with nuclear chromatin condensation increased significantly from 18–22 h (p = 0.003). Likewise, the proportion of ookinetes with fragmented DNA rose significantly between 18 and 22 h (p = 0.0034). Although the proportion of TUNEL positive cells was expected to be similar to those positive for acridine orange and CaspaTag, a higher proportion was observed (acridine orange: 34.5 ± 1.76%, CaspaTag: 30.15 ± 2.14% and TUNEL: 48.55 ± 6.01% respectively at 18 h), however in this analysis no account was taken of potential false positive results that we showed to be associated with this assay. Compared to the other markers, annexin positive ookinetes remained at a lower level (19.57 ± 1.88%, 28.33 ± 5.61% and 30.12 ± 2.75% at 18, 22 and 26 h respectively) throughout the study, but these values exclude and ookinetes with both annexin staining and compromised membranes. A further analysis was performed (a 2 way AVOVA) in which data from the TUNEL assay were adjusted to take account of the expected false positive results by subtracting 10% from each value and in addition, the proportion of ookinetes that were annexin and propidium iodide positive were added to the proportion displaying annexin alone (ie. The total proportion of ookinetes displaying annexin labeling). When data were analysed in this way there was no significant difference between the expression of any of the markers of apoptosis at any time point(F_(3,24) _= 2.16: *p *= 0.119) thus a similar proportion of ookinetes displayed each of the markers of apoptosis at each time point.

**Table 1:** Summary of the proportion of ookinetes expressing four different markers of apoptosis at three time points post-incubation of gametocytaemic mouse blood.

Time (hrs)	Condensed chromatin	Caspase-like activity	Fragmented DNA	PS translocation	PS + permeable membrane	Permeable membrane
18	34.5 ± 1.76	30.15 ± 2.14	48.55 ± 6.01	19.57 ± 1.88	13.25 ± 0.689	14.88 ± 2.56

22	55.80 ± 13.68	43.49 ± 1.53	64.19 ± 6.09	28.33 ± 5.61	13.66 ± 2.79	19.55 ± 4.29

26	49.01 ± 5.51	47.72 ± 3.93	69.89 ± 2.81	30.12 ± 2.75	14.67+0.519	19.40+2.73

Each value is a mean of 3 experiments, n = 150 ookinetes for each experiment. PS = phosphatidylserine.

## Discussion

The hypothesis that apoptosis-like cell death occurs in *Plasmodium *species has been challenged as a result of conflicting results observed by different research groups. With respect to *P. falciparum*, differences in sensitivity to chloroquine treatment between different parasite strains has been suggested to be at the heart of these differences [26]. Treatment of chloroquine sensitive (clone 3D7) *P. falciparum *with the anti-malarial drug, chloroquine, was shown to induce DNA fragmentation *in vitro*, loss of mitochondrial membrane potential and DNA fragmentation [26-28]. More evidence of markers such as nuclear chromatin condensation, caspase-like activity and loss of mitochondrial membrane potential were also reported in erythrocytic stages in *P. falciparum *isolate NF54 treated with bilirubin [29]. However, Deponte and Becker [22] and Nyakeriga *et al *[30] reported that the chloroquine sensitive F32 strain of *P. falciparum *die by a non-apoptotic cell death because they found no evidence for nuclear DNA fragmentation or externalisation of PS to the outer surface of the plasma membrane. Further, a chloroquine-resistant *P. falciparum *isolate, PSS1, showed no nuclear chromatin condensation or caspase-like activity, although a very low percentage of TUNEL positive (5–9%) parasites and cytoplasmic vacuolation was observed [21].

With respect to *P. berghei*, the observation of multiple features of apoptosis-like cell death in ookinetes reported by Al'Olayan et al. [23] was not confirmed by Le Chat and colleagues (2007), using the same clone of *P. berghei*. They were unable to find evidence of ookinetes with condensed chromatin or fragmented DNA although they did detect a very small proportion (~3%) of parasites displaying PS translocation and a similar proportion with activated caspase-like activity after 21 h of culture which, in the case of the latter, rose to ~14% at 24 h post-culture.

Here we report that features of metazoan apoptosis, such as nuclear chromatin condensation, activation of caspase-like molecules, nuclear DNA fragmentation and translocation of phosphatidylserine to the outer surface of the plasma membrane were again observed in *P. berghei *ookinetes that had been grown in well-established culture conditions [31], and when incubated further in PBS alone. In addition, we demonstrated that MOMP occurred in approximately one third of *P. berghei *ookinetes after incubating gametocytaemic blood for 18 h.

Although apoptosis-like cell death has been observed in the blood cell stages of *P. falciparum *this is the first report of DNA fragmentation in *P. falciparum *ookinetes that had developed in the mosquito midgut, suggesting that this type of PCD is not confined to ookinetes of a rodent malaria parasite.

Mitochondria play a key role in the induction of apoptosis in mammalian cells with the release of cytochrome c and subsequent caspase activation following MOMP (e.g. [32,33]. However MOMP is not associated with apoptotic pathways in all metazoans and the degree to which it is conserved remains to be determined [34]. Reports that MOMP and cytochrome c release occur during apoptosis induction in the trypanosomatids [2,19] and that chloroquine was shown to induce a significant Δψ_m _collapse in a dose-dependent manner in *P. falciparum *[26] suggest it may be part of an ancient cell suicide/survival mechanism [35,36]. A link between MOMP and downstream apoptotic pathways in *Plasmodium *still needs to be established.

In common with other protozoan parasites, a proportion of *P. berghei *ookinetes react positively with a general caspase inhibitor (eg. 17.0 ± 2.12% at 18 h). This proportion varies from experiment to experiment but generally increases with time of incubation. Caspase-like activity correlated well with another apoptosis marker, chromatin condensation, when mature ookinetes were further incubated in PBS (eg.15.5 ± 1.06 at 18 h). The absence of caspases from protozoan genomes calls into question the target of so called caspase-specific inhibitors [16] whose specificity has been challenged [37]. Caspases belong to the clan CD cysteine proteases, as do the metacaspases [38] which are under scrutiny as homologous proteases that may be involved in apoptosis in unicellular eukaryotes (e.g. [26,39,40]). Meslin and colleagues [26] propose a putative role for metacaspases in apoptosis in *P. falciparum *but no evidence supports this hypothesis with respect to *P. berghei *[25], and, in our hands, ookinetes in which the gene for the metacaspases *PbMC1 *[25] or *PbMC2 *had been disrupted exhibited apoptosis markers to the same degree as the wild-type (Arambage and Hurd, in preparation). Attempts to identify the cysteine protease(s) that is binding to proprietary caspase inhibitors or being inhibited by a range of cysteine protease inhibitors [23] are ongoing. At present the involvement of the molecule(s) binding to caspase-inhibitors in pathways leading to apoptosis-like cell death in *P berghei *ookinetes is speculative but the close agreement in the proportion of ookinetes displaying this marker and condensed chromatin and DNA fragmentation over time is supportive of some connection between their action and PCD.

**Figure 9:**
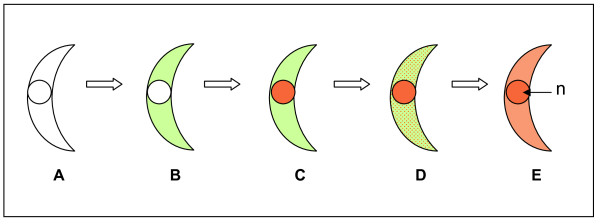
**schematic for the proposed progress of apoptotic-like cell death in *P. berghei *ookinetes**. A: Live ookinete with no caspase activity (no staining) B: Apoptotic ookinete with caspase-like activity (green), C and D: Ookinete with caspase-like activity and a compromised cell membrane (late stage apoptosis) (green and orange), E: ookinete with no caspase-like activity but with a compromised membrane (orange) (may have died by necrosis), n: nucleus; green: the fluorochrome labelled caspase inhibitor (FAM.VAD fmk); orange: propidium iodide.

In this study, many ookinetes that had developed in culture were shown to have lost their outer membrane integrity. Although the nature of the assays for condensed chromatin and fragmented DNA make it impossible to simultaneously determine the membrane integrity in ookinetes displaying these markers of apoptosis, we were able to do so in the assays to detect PS translocation and the activation of caspase-like molecules. In these cases, we found a proportion of parasites displaying the marker for apoptosis also had a compromised cell membrane. Loss of membrane integrity has been associated with late stages of apoptosis in mammalian cells, known as the necrotic phase of apoptosis [41], and has been recorded following induction of apoptosis in the protozoan parasites *Leishmania *spp. and *T. cruzi *(reviewed in [19]). As it was not possible to follow the progress of the appearance of apoptotic features in an individual ookinete, we are not able to verify the timing of the loss of membrane integrity relative to other markers but propose that, here too, this may occur late in the process of dying (See Fig. 9). Interestingly, Le Chat and colleagues [25] also reported that many ookinetes staining positive for PS translocation and caspase-like activity also had compromised membranes. This observation left us with a dilemma: how to classify ookinetes that displayed markers for apoptosis but also had compromised membranes? This was particularly problematical for detection of PS translocation by annexin labelling. Ookinetes that were annexin and PI positive could have reached a late stage of apoptosis. Alternatively, a compromised membrane may admit annexin into the cell thus staining PS that had not been translocated. Therefore, ookinetes with both annexin and propidium iodide staining could be regarded either as late apoptotic or as having died by some other process. Annexin labelling was not seen in all cells stained with PI suggesting the former is more likely than the latter. In the analysis of our time-course studies we initially erred on the side of caution and only included ookinetes with intact membranes in the data sets where PI staining was also conducted. This may explain why a higher proportion of ookinetes appear positive for condensed chromatin and fragmented DNA than the other apoptosis markers, as these counts could include ookinetes with compromised membranes.

TUNEL assays are routinely used to detect fragmented DNA associated with apoptotic cells. The histochemical TUNEL assay used in this study relies upon a detection system based on streptavidin-horseradish peroxidase. Our control for non-specific staining suggested that approximately 10% of ookinetes scored positive may be false positives. This would be an additional explanation for the apparent detection of a higher proportion of ookinetes positive for DNA fragmentation than other markers of apoptosis. Recent work, using an in situ death detection kit fluorescein to detect fragmented DNA (Roche Diagnostics Ltd) indicates that this would be a more useful assay for use with ookinetes as it is quick to perform, the fluorescence label enables more rapid detection of apoptotic ookinetes and false positives are unlikely to occur (personal communication, Pollitt and Reece).

Our second analysis included all ookinetes that were positive for the respective markers, and excluded a fixed proportion of ookinetes that our analysis showed likely to be false positives for TUNEL staining. This demonstrated that each marker was displayed by a similar proportion of the population, thus suggesting that the morphological and biochemical markers may all be linked in a process with an end result similar to apoptosis in metazoans,

Deponte and Becker [22] suggested that methodological pitfalls associated with different assays could cause differences in the detection of markers of apoptosis. We found it necessary to adapt apoptosis assays primarily designed for use with mammalian cell lines. Incubation temperature was particularly important as sexual stages of *P. berghei *are highly temperature sensitive and produce no midgut and salivary gland infections at 28°C or above [42], necessitating the routine culture of *P. berghei *ookinetes at 19–20°C. Differences in incubation temperatures, reagent concentrations or the use of kits from different manufacturers may explain the inconsistency of results obtained by different laboratories. We recommend the use of several markers in future experiments on apoptotic-like cell death in ookinetes.

The majority of work reported here was performed using *P. berghei *ookinetes that had developed *in vitro*. We wished to determine whether ookinetes of the human malaria, *P. falciparum *were also capable of undergoing apoptosis. Although *P. falciparum *ookinetes have been produced in culture [43], this is not a technique that is routinely successful. We therefore investigated *P. falciparum *ookinete death *ex vivo*. In common with *P. berghei *ookinetes taken from the mosquito midgut lumen [23], we observed that a large proportion (~68%) of *P. falciparum *ookinetes were dying by an apoptosis-like process 24 h after the ingestion of gametocytes by mosquitoes, This strengthens our hypothesis that conditions in the midgut lumen are ultimately unfavorable for ookinete survival and a large proportion of the losses that occur between ookinete production and oocyst formation are the result of cell suicide, and take place before the ookinete traverses the midgut epithelial cells [16].

Having examined ookinetes that develop from blood from a number of different mice we have become aware of large variations in the proportion of ookinetes displaying signs of apoptosis at the same age (eg. nuclear chromatin condensation and caspase-like activity at 18 h varied from 15–35% and 15–30% respectively), and also a difference in the increase in this proportion as the ookinetes age. In particular, we observed that at 18 h post-blood collection onwards those ookinetes from mice with parasitaemias above 10% develop compromised membranes more quickly. At 18 h a higher proportion of ookinetes from mice with asexual parasitaemias below 10% display markers of apoptosis thus it is likely that many parasites from highly infected mice have already progressed to late stages of apoptosis or are dead by 18 h (data not shown). It is possible that factors present in the blood of mice with high asexual parasitaemia act as triggers to induce ookinetes to die by an apoptosis-like pathway.

The proportion of apoptotic ookinetes rose more rapidly with time when incubated in PBS, than when ookinetes remained in the nutrient rich ookinete medium. This may be because there was a higher proportion of apoptotic ookinetes at the start of monitoring this experiment (18 h) compared with the previous one. Alternatively, the lack of nutrients in PBS, may have caused starvation-induced apoptosis, as occurs in *D. discoideum *[44] and *T. cruzi *[1]. In contrast to the latter studies, Jimenez *et al*. [45] report that serum deprivation induces autophagic characteristics such as monodansylcadaverine-labeled vesicle accumulation in *T. cruzi*. Menna-Barreto et al. [46] also reported autophagosome formation in *T. cruzi*, this time under drug pressure, and suggest that evidence indicative of apoptosis, autophagy and necrosis in *T. cruzi *are suggestive of cross talk between these three death pathways. The observation of cytoplasmic vacuolation without evidence of DNA fragmentation when a chloroquine-resistant isolate of *P. falciparum *red blood cell stages was subjected to drug and nitric oxide pressure led Totino and co-authors [21] to conclude that these parasites were dying by autophagy rather than apoptosis. In our study, we occasionally saw vacuolation in ookinetes at late stages of apoptosis.

Ookinetes display the majority of Clarke's markers for type 1 programmed cell death or apoptosis [47]. However it is becoming clear that pathways involved in cell death are not clear cut and that these processes are interlinked [18]. With respect to unicellular eukaryotes, trying to shoehorn observations into classifications designed for multicellular organisms is clearly not the way forward. Some form of non-necrotic death is taking place in these organisms but, although outward signs are similar, molecular pathways clearly differ as homologues of apoptosis-inducing factors and effector caspases regulating apoptosis in metazoans have not been found [17]. The evolution of cell suicide mechanisms evidently predates the metazoans [37]. We now need to search for novel induction and regulation pathways as well as explanations for a phenomenon that likely has a different function to that in the higher eukaryotes.

## Conclusion

A proportion of *P. berghei *and *P. falciparum *ookinetes do show markers similar to mammalian cells that are undergoing apoptosis. These markers are expressed without experimental exposure to drugs or other stressors and must be a result of conditions present both in culture and *in vivo*. Several markers are displayed by a similar proportion of *P. berghei *ookinetes and this proportion increases with time. Work is underway to identify the triggers that induce this cell suicide.

## Materials and methods

### Ookinete growth and enrichment

All experiments were carried out in accordance with the UK Animals (Scientific Procedures) Act 1986 using approved protocols. CD mice were infected with *P. berghei *ANKA 2.34 and gametocytaemic blood collected by cardiac puncture and incubated as described in Carter et al. [48] in ookinete medium composed of supplemented RPMI. Fertilized gametocytes developed into retort forms and then ookinetes in this medium. At various times from 16 h post-incubation onwards, ookinetes were purified by separation from erythrocytes using a MidiMacs (Miltenyi Biotec, Germany) separation column as described in Carter et al. [48,49] and used to assess the proportion that exhibited markers of apoptosis-like cell death.

### Markers of apoptosis

Samples of ookinetes from the same culture were divided according to the experimental protocol and each sub-sample subjected to a different assay to identify different signs of programmed cell death as follows:

### Detection of nuclear chromatin condensation

Nuclear chromatin condensation was detected by staining ookinetes suspended in 5 μl PBS with an equal volume of acridine orange (Sigma) at a working concentration of 2.5 μg/ml in PBS. After addition of ~0.5 μl of Vectashield mounting medium (Vector Laboratories Inc., Buringame, Canada), samples were immediately viewed under oil immersion (×1000) using a Leica inverted microscope (Leica Microsystems, Germany) with a filter suitable for fluorescein (450/490 excitation; 515 emission). The number of ookinetes with condensed chromatin out of one hundred from each sample was counted (unless otherwise specified). Results are expressed as % of ookinetes exhibiting positive staining.

### Detection of activated caspase-like molecules

Active caspase-like molecules were detected using the CaspaTag fluorescence caspase activity kit (Chemicon international, USA) which contains the caspase inhibitor, carboxyfluorescein-Val-Ala-Asp fluoromethylketone (FAM.VAD.fmk) which binds irreversibly to activated caspases in 1:1 stoichiometry, thus detecting apoptotic cells. Caspase-like activity was also detected with sulphorhodominyl-Asp-Gly-Val-Asp-fluoromethylketone (SR.DEVD.fmk) but this was not used for time course studies. Propidium iodide (PI) (250 μM/mL) was used in some of these assays to detect cells with permeabilised membranes. Assays were performed according to the manufacturer's instructions; except that incubations were carried out at 19°C as a pilot study had shown that the plasma membrane of ookinetes incubated at 37°C rapidly became permeable to propidium iodide (PI). After addition of 0.5 μl of Vectashield samples were observed under oil immersion (×1000) with a filter suitable for fluorescein or rhodamine (515–560 excitation; 580 emission) and the proportion of ookinetes with active caspase-like molecules or signs of PI infiltration were recorded.

### DNA fragmentation assay

DNA fragmentation was detected by TdT-FragEL™ DNA fragmentation detection kit (TUNEL) (Calbiochem, UK). The assay is based on binding of terminal deoxynucleotidyl transferase (TdT) to exposed 3'-OH ends of DNA fragments, generated due to apoptotic signals. Ookinetes were first fixed in 4% methanol-free paraformaldehyde (Polyscience Inc. Warrington, PA) for 10 mins and resuspended in 80% ethanol at a cell density of 1 × 10^5^/ml. Approximately 10 μl of the suspension was dried onto each well of a multi-well slide and the assay was performed on the fixed ookinetes according to the manufacturer's instructions. Samples were observed under bright light at ×1000 magnification. The position and shape of apoptotic nuclei were confirmed by staining Vectashield containing 4',6-diamidino-2-phenylindole (DAPI) (Vector Laboratories, Canada).

Since the TUNEL reaction is based on a biotin-streptavidin-DAB detection system, a negative control was performed by removing TdT enzyme from the labelling reaction mixture to detect any non specific binding to endogenous peroxidases, resistant to the inactivation step in the protocol and acting on DAB to create the coloured product. Three replicates from the same sample at each time point were subjected to TUNEL assay using this negative control and compared with a sample assayed using the complete reaction.

### Translocation of phosphatidylserine to the outer leaflet of the plasma membrane

This was detected using an Annexin V-FITC apoptosis detection kit (Sigma, UK), which detects translocation of phosphatidylserine (PS), in the presence of Ca^2+^. The assay was conducted according to the manufacturer's instructions except that incubations were performed for 12 mins at 19°C. One microlitre of PI (as provided by the kit manufacturers) was added and the sample incubated again for 3 mins. After addition of 0.5 μl of Vectashield, samples were immediately observed using a FITC filter at ×1000. In cells regarded as apoptotic, the outer margin of the ookinete was labelled bright, apple green and PI was excluded.

### Mitochondrial trans-membrane potential assay

Mitochondrial depolarisation was measured by a JC-1 assay kit (Molecular Probes, UK). This includes a cationic dye which is known as 5,5',6,6'-tetrachloro-1,1',3,3' tetraethylbenzimidazolylcarbocyanine iodide (JC-1). JC-1 shows a potential-dependent accumulation in mitochondria. Loss of Δψ_m _can be detected by the shifting of emission of fluorescence from orange (polarised mitochondrial membrane) to green (depolarised mitochondrial membrane). The assay was performed according to the manufacturer's instructions using ~1 × 10^6 ^ookinetes/1 ml. *P. berghei *ookinetes were enriched from ookinete culture medium at 18 h and suspended in PBS. Each sample of 100 μl of ookinete suspension was then incubated with 1 μl of 200 μM JC-1 for 15–30 mins at 19°C. Cells were washed once in PBS. After addition of 0.5 μl of Vectashield, they were immediately observed using a FITC filter.

### A temporal profile of the expression of apoptosis markers *in vitro*

The effect of age on the proportion of ookinetes undergoing apoptosis was examined under two different culture conditions.

#### Incubation in phosphate buffered saline

Blood samples obtained from different mice were incubated in ookinete medium at 19°C for 17 h. Ookinete cultures were pooled and ookinetes purified, then pelleted, and resuspended in 2 ml PBS at an unknown density which varied from experiment to experiment, and incubated at 19°C. A temporal profile of ookinetes exhibiting markers of apoptosis was constructed by removing aliquots from the ookinete suspension at 18, 20, 22, 24, 26 h post-initial culture, and stained using acridine orange or CaspaTag as before. The total number of apoptotic ookinetes and dead cells/100 ookinetes was detected under oil immersion, ×1000 magnification, and the experiment was repeated twice.

#### Incubation in ookinete medium

Gametocytaemic *P. berghei *blood samples were incubated at 19°C in ookinete medium for 18, 22 and 26 h. Ookinetes were then assessed for 4 markers of apoptosis using 4 different assays namely; acridine orange, CaspaTag, Annexin V and the TUNEL assay. A proportion of ookinetes in the samples showed markers of apoptosis and had permeabilised membranes at the same time. With the exception of Annexin V labeling, we regarded these as late apoptotic ookinetes. The total number of apoptotic, late apoptotic and dead cells/150 ookinetes per sample were detected. The experiment was repeated three times.

### Detection of apoptosis in ookinetes of Plasmodium falciparum

As no reliable method for the development of *P. falciparum *ookinetes in culture was available to us, ookinetes were examined *ex vivo *to determine whether a proportion of this *Plasmodium *species also showed signs of apoptosis-like death. *P. falciparum *clone 3D7 was cultured *in vitro *using conditions permissive for the development of mature gametocytes infective to mosquitoes [50-52].

*An. gambiae *Keele strain mosquitoes were maintained in an insectary at 26 ± 1°C at 70–80% relative humidity in a 12:12 hour light/dark cycle. Adult mosquitoes were given *ad libitum *a 5% solution of 5% glucose/0.05% para-amino-benzoic acid, except for 24 h prior to a blood feeding; only distilled water was provided at this time.

Five to seven days after emergence from pupae, adult female *An. gambiae *were membrane-fed on uninfected human blood mixed with cultured asexual and gametocyte stages of *P. falciparum *according to standard procedures. Twenty four hours post-infection mosquitoes were killed and guts removed immediately into 40 μl PBS. 10 μl was diluted further with 30 μl or 70 μl PBS and then 10 μl spotted on to a multi well slide. This gave 10 μl spots with 1/16^th ^or 1/32^nd ^of a midgut, depending on the dilution used. The slides were then air dried and fixed using alcohol free 4% formaldehyde, and then frozen at -80°C until subjected to a TUNEL assay.

### Statistical analysis

All data were analysed using Mintab^® ^software (version 13.1). Initially data was checked for normality (Anderson-Darling normality test) and equal variance (Bartlett's test) before analysis. Data that were normally distributed were then analysed using 2-way ANOVA, non-normally distributed data were first arcsin converted. Statistically significant data were further analysed using Tukey's test for paired comparisons. The effect of temperature on caspase activity and viability of ookinetes was analysed using a 2 sample t-test.

## Competing interests

The authors declare that they have no competing interests.

## Authors' contributions

SCA contributed to the design of the study, carried out all the assays for apoptotic markers and statistical analysis in the *P. berghei study *and assisted in drafting the manuscript. IP carried out the analysis of apoptosis in *P. falciparum *ookinetes. LR-CL contributed to the design of the study of *P. falciparum*, prepared and fixed the ookinetes, and assisted with drafting of the manuscript. KG contributed to the design of the study and to the manuscript draft. HH conceived of the study, and participated in its design and coordination and helped to draft the manuscript. All authors read and approved the final manuscript.
